# Septic arthritis of the knee: the use and effect of antibiotics prior to diagnostic aspiration

**DOI:** 10.1308/003588412X13171221591015

**Published:** 2012-07

**Authors:** P Hindle, E Davidson, LC Biant

**Affiliations:** NHS Lothian,UK

**Keywords:** Septic arthritis, Antibiotics, Aspiration, Knee

## Abstract

Septic arthritis of the native knee joint and total knee arthroplasty both cause diagnostic and treatment issues. There is no gold standard test to diagnose a joint infection and the use of joint aspiration is commonly relied on. It is widely accepted by orthopaedic surgeons that antibiotics should be withheld until aspiration has been performed to increase the odds of identifying an organism. Patients often present to other specialties that may not be as familiar with these principles.

Our study found that 25 (51%) of the 49 patients treated for septic arthritis of the native or prosthetic knee in our unit over a 3-year period had received antibiotics prior to discussion or review by the on-call orthopaedic service. Patients were significantly less likely to demonstrate an organism on initial microscopy (entire cohort: *p*=0.001, native knees: *p*=0.006, prosthetic knees: *p*=0.033) or on subsequent culture (entire cohort: *p*=0.001, native knees: *p*=0.017, prosthetic knees: *p*=0.012) of their aspirate if they had received antibiotics. The sensitivity of microscopy in all patients dropped from 58% to 12% when patients had received antibiotics (native knees: 46% to 0%, prosthetic knees: 72% to 27%). The sensitivity of the culture dropped from 79% to 28% in all patients when the patient had received antibiotics (native knees: 69% to 21%, prosthetic knees: 91% to 36%).

This study demonstrated how the management of patients with suspected cases of septic arthritis of the knee may be compromised by empirical administration of antibiotics. These patients were significantly less likely to demonstrate an organism on microscopy and culture of their initial aspirate. There is a significant high false negative rate associated with knee aspiration with prior administration of antibiotic therapy.

Septic arthritis of the knee joint is associated with significant morbidity and mortality.^1–5^ The incidence of septic arthritis varies from 2 to 10 per 100,000 in the general population.^6^ In one series the incidence of septic arthritis of native joints was reported to be 9.2 per 100,000 with more than half of these cases involving the knee.^7^ Over 6,000 primary knee replacements are performed in Scotland each year with 1.6% of patients requiring readmission for superficial or deep infection.^8^ The reported rates of infection are 0.6–2% in England and Wales.^9^ Together, native knee joints and total knee replacements represent a significant number of patients presenting with septic arthritis of the knee. Patients with an infection of a native knee are a distinct population to those with an infection of their total joint replacement despite similar initial methods of investigation and treatment.

Septic arthritis can be defined as the invasion of a joint by an infectious agent. This is most commonly by bacteria but can also be caused by viral, mycobacterial or fungal agents. Infections around the knee joint can either be considered superficial (external to the joint) or deep (involving the joint). Septic arthritis is accepted as pertaining to a deep infection. The main problem with research looking at septic arthritis is that there is no accepted gold standard test that can identify an infected joint.

Treatment of septic arthritis of the knee involves prompt recognition and diagnosis, isolation of the responsible organism, early surgical lavage, debridement and prolonged treatment with targeted antibiotics.^4^ This initial process is similar for patients with or without a total joint replacement. Patients with early acute post-operative infection of their total knee replacement may undergo radical debridement and retention of the components with exchange of the polyethylene insert.^10,11^ Patients may require a single or two-stage revision of their prosthesis if they develop a chronic deep infection.^11,12^

Acute post-operative septic arthritis of a joint replacement diagnosed early has a more favourable prognosis compared with infection in a well established prosthesis due to haematogenous spread.^13,14^

It is widely accepted by orthopaedic surgeons that the use of antibiotics in suspected cases of septic arthritis should be withheld until aspiration of the joint can be performed.^7^ Antibiotics reduce the possibility of isolating an organism and this prevents targeted antibiotic treatment of the infection following lavage. In some cases aspiration is bypassed and samples are directly collected at the time of joint lavage.^9^

The majority of patients present initially to non-orthopaedic surgeons (primary care, emergency medicine), where these principles may not be adhered to. An initial audit of patients admitted to our unit showed that there was a large number of patients receiving antibiotics prior to discussion with or review by the orthopaedic service. The aim of this study was to examine the effect of prior antibiotic administration on the ability to identify the responsible organism in patients with septic arthritis of the native and prosthetic knee.

## Methods

A retrospective analysis was undertaken of patients admitted to the study centre with septic arthritis of the knee between January 2006 and June 2009. There is no single pathognomonic sign or test to diagnose septic arthritis. Organisms on culture can represent contamination from the skin and material that looks like pus can represent a non-septic arthropathy. Other studies have had the same problem and have used a mixture of definitions. These include arthritis with a positive aspiration or joint fluid culture, a strong clinical suspicion or a definite radiological diagnosis.^1,2^

Our study group comprised patients with a clinical diagnosis of septic arthritis based on the presenting history, examination, blood test results and knee aspirate. Only patients who underwent a full course of treatment for septic arthritis by the attending consultant were included in the study. Any cases where the microbiologists commented that contamination was likely were excluded. All superficial infections, non-infective inflammatory arthritides or crystal arthropathies were also excluded. These criteria ensured that all cases represented a true septic arthritis.

The results of the initial aspirate were recorded. These data were split into the appearance of the fluid, identification of an organism on microscopy and Gram staining, and the presence of any crystals. The number of white cells per field was not available in our unit. The fluid was cultured on blood and chocolate agars with an extended broth culture. Samples were not routinely checked for tuberculosis or fungi. Isolation of organisms that were cultured subsequently on initial or broth cultures and any associated sensitivities were recorded.

The patient demographics recorded included age, sex, diabetes, steroid use, presence of pre-existing knee pathology, previous surgery and time elapsed since their previous surgery. Admission temperature, white cell count, neutrophil count, C-reactive protein (CRP) levels and erythrocyte sedimentation rate (ESR) were recorded. The results of microbiology samples obtained after the original specimen were also noted.

After their initial aspirate, all patients underwent either an open or arthroscopic washout. Total joint replacements also underwent exchange of the polyethylene insert when possible. Multiple further samples were routinely taken for microbiology analysis at operation.

A Kolmogorov–Smirnov test was used to ascertain normality of the data sets. Mann–Whitney U, one-tailed Pearson chi-squared and Fisher’s exact tests were used for statistical analysis. All data analysis was carried out using SPSS® version 19 (SPSS, Chicago, IL, US). A *p*-value of <0.05 assumed significance.

## Results

There were 49 consecutive patients eligible for this study (36 men and 13 women). There was a bimodal distribution for age with peaks at 30 and 70 years of age. There were 22 patients with an infection of their total joint prosthesis and 27 with an infection of their native knee joint. In the prosthetic group, two had had recent implantation (fewer than 258 days prior to presentation). The rest were well established prostheses with no prior signs of infection recorded or initial surgical complication. Of the patients with native knee infections, seven had either had surgery or open trauma (with no associated bony injury) to the knee in the previous six weeks. These cases are shown in Table 1 with the time from surgery or trauma to their presentation with an infection.

**Table 1:** Recent surgical procedures or trauma in native knee infections

Patient	Procedure	Time elapsed
1	Lateral meniscectomy	3 days	2	Steroid injection	7 days	3	ACL reconstruction	6 weeks	4	Quads tendon repair	1 day	5	ACL reconstruction	5 days	6	ACL reconstruction	3 weeks	7	Primarily closed traumatic wound	1 week

ACL = anterior cruciate ligament

The majority of patients (*n*=21, 43%) presented initially to the emergency department. The rest presented to primary care (*n*=15, 31%), physicians (*n*=8, 16%) and orthopaedic surgeons other than the acute receiving team (*n*=5, 10%). Twenty-five patients (51%) had received antibiotics prior to having an aspirate of their knee. The antibiotics were prescribed by general practitioners (*n*=12, 48%), emergency medicine doctors (*n*=7, 28%) and physicians (*n*=6, 24%). Those who had received antibiotics had a delay to review by the orthopaedic service (7 vs 4 days from initial presentation) but this did not reach statistical significance (*p*=0.054).

Seventeen patients (35%) demonstrated organisms on initial microscopy and Gram staining. This represented 3 of the 25 (12%) who had received antibiotics and 14 of the 24 (58%) who had not. Patients were significantly less likely to demonstrate an organism on their initial microscopy if they had antibiotics prior to aspiration (*p*=0.001).

The joint fluid of 26 patients (53%) demonstrated an organism on culture, an extra 9 cases in addition to those with an organism identified on microscopy of the initial aspirate. This represented 7 of the 25 (28%) who had received antibiotics and 19 of the 24 (73%) who had not. Patients were significantly less likely to culture an organism from their aspirate if they had antibiotics prior to aspiration (*p*=0.001).

There were two acute post-operative infections following total knee replacement. In the native knees there were seven cases with either penetrating trauma or recent surgery. Due to the small numbers in these subgroups, further subgroup analysis was not performed on these cases.

### Patients with septic arthritis of a native knee joint

Fourteen out of twenty-seven cases (52%) had received antibiotics prior to aspiration. Analysis of the patients with a native joint infection showed 6 of the 27 (22%) demonstrated an organism on microscopy and Gram staining. All of these cases were in patients not receiving antibiotics. Patients were more likely to demonstrate an organism on microscopy of their aspirate if they had not received antibiotics (*p*=0.006). Twelve (44%) grew an organism on culture; three had received antibiotics and nine had not. Patients were more likely to grow an organism on culture of their aspirate if they had not received antibiotics (*p*=0.017).

### Patients with septic arthritis of a prosthetic joint

Eleven of the twenty-two cases (50%) had received antibiotics prior to aspiration. Analysis of the patients with a prosthetic knee joint infection showed that 11 of the 22 (50%) demonstrated an organism on microscopy and Gram staining. Three of these cases had received antibiotics and eight had not. Patients were more likely to demonstrate an organism on microscopy of their initial aspirate if they had not received antibiotics (*p*=0.033). Fourteen (64%) grew an organism on culture; four had received antibiotics and ten had not. Patients were more likely to grow an organism on culture if they had not received antibiotics (*p*=0.012).

The sensitivities of microscopy and culture of the aspirate are summarised in Table 2. These are shown for the entire cohort and also for both the native and prosthetic knee groups. These data demonstrate that there is a significant drop in the sensitivity of the aspirate with the administration of antibiotics in all groups.

**Table 2:** Sensitivities of aspiration

Group	Test	No antibiotics	Antibiotics
All cases	Microscopy	58%	12%
	Culture	79%	28%
No prosthesis	Microscopy	46%	0%
	Culture	69%	21%
Prosthetic joint	Microscopy	72%	27%
	Culture	91%	36%

Initial (tympanic membrane) temperatures of patients were recorded on presentation to secondary care. The admission white cell count, neutrophil count, CRP levels (normal value: 0–5mg/l) and the ESR (normal value: 3–15mm/hr) were also noted (Table 3). Patients who had received antibiotics were significantly more likely to have lower white cell and neutrophil counts (*p*=0.002 and *p*=0.017 respectively). The data for prosthetic and native joints are shown in Table 4. Patients with a native knee infection who had received antibiotics were significantly more likely to have lower white cell and neutrophil counts (*p*=0.013 and *p*=0.026 respectively). This was not the case in patients with a prosthetic knee infection (*p*=0.083 and *p*=0.225 respectively).

**Table 3:** Recorded variables in the entire cohort

Variable	All cases	Received antibiotics	Did not receive antibiotics	*p*-value
Temperature	37.7ºC	37.5ºC	37.8ºC	0.252
White cell count	12.0 × 10^9^/l	10.1 × 10^9^/l	14.0 × 10^9^/l	**0.002**
Neutrophil count	9.9 × 10^9^/l	8.0 × 10^9^/l	11.1 × 10^9^/l	**0.017**
C-reactive protein	189mg/l	177mg/l	202mg/l	0.610
Erythrocyte sedimentation rate	70.8mm/hr	81.4mm/hr	62.1mm/hr	0.274

**Table 4:** Recorded variables split by knee type

Variables	Non-prosthetic joints			Prosthetic joints		
	Antibiotics	No antibiotics	*p*-value	Antibiotics	No antibiotics	*p*-value
Temperature	37.7ºC	38.1ºC	0.264	37.4ºC	37.5ºC	0.734
White cell count	10.3 × 10^9^/l	13.6 × 10^9^/l	**0.013**	10.0 × 10^9^/l	14.8 × 10^9^/l	0.773
Neutrophil count	7.3 × 10^9^/l	10.4 × 10^9^/l	**0.026**	8.4 × 10^9^/l	12.0 × 10^9^/l	0.083
C-reactive protein	179mg/l	207mg/l	0.778	175mg/l	193mg/l	0.225
Erythrocyte sedimentation rate	78.3mm/hr	66.4mm/hr	0.549	83.6mm/hr	55.9mm/hr	0.354

In the prosthetic knee group, nine patients (41%) underwent surgical lavage with polyethylene spacer exchange and five (23%) eventually underwent revision of all components. The reason for some patients not having a polyethylene spacer exchange was due to treating patients from other hospitals whose implants were not kept in stock and treatment delay was not appropriate. There was no relation to previous use of antibiotics and the need for subsequent revision (*p*=0.17).

The most frequent responsible organisms were staphylococci or streptococci. There were also *Escherichia coli*, *Pseudomonas*, anaerobes and one case of methicillin resistant *Staphylococcus aureus*. These responsible organisms and their antibiotic sensitivities are shown in Table 5.

**Table 5:** Isolated organisms and associated antibiotic sensitivities

Cultured organisms	Number of cases	Antibiotic sensitivities
*Staphylococcus aureus*	12	Flucloxacillin, erythromycin
*Streptococcus* spp	8	Penicillin
Coagulase-negative *Staphylococcus*	2	Doxycycline, rifampicin, vancomycin
*Escherichia coli*	1	Ciprofloxacin, amoxicillin, gentamicin
MRSA	1	Vancomycin, rifampicin, clindamycin
*Pseudomonas*	1	Ciprofloxacin
Anaerobic cocci	1	Metronidazole

## Discussion

This study highlights the problem and consequences of patients receiving antibiotics prior to samples being taken for microbiology analysis. Over half the patients admitted to our unit with septic arthritis of the knee had already received antibiotics. These patients were significantly less likely to demonstrate an organism on initial microscopy or culture of their aspirate.

Forty-two per cent of patients had a negative result from microscopy of their initial aspirate (native joints 54% and prosthetic joints 28%). This demonstrates that an initial aspirate alone is not sensitive enough to exclude a septic arthritis and cannot be relied on in isolation. Administration of antibiotics significantly decreases the sensitivity even further. Clinicians should elicit an accurate drug history of recent antibiotic use as this will highlight that the aspirate might be even less likely to yield a true positive result.

There was a trend to having a lower temperature or inflammatory blood marker if the patient had received antibiotics. This information alone will not help differentiate between those who do or do not have a septic arthritis but it needs to be taken into account when considering the entire clinical picture. None of the patients in our series of septic arthritis cases had normal CRP levels.

The diagnosis of septic arthritis is not always clear and one of the problems with research addressing this issue is establishing which cases can be assigned a diagnosis of septic arthritis of the knee with certainty. This has been a limitation of both the present and other studies.^2,3,7,9,15^ There is currently no gold standard for diagnosis. The stringent inclusion criteria of our study should have excluded any cases that were not a true septic arthritis of the knee and would therefore have limited any false positives. We recognise that there may be a group of patients who will not reach secondary care who have had superficial infections treated successfully in primary care with a course of oral antibiotics.

Our results demonstrate that joint aspiration needs to be performed in a targeted fashion and with knowledge of the associated low sensitivity rate, particularly in those who have received antibiotics. If the clinician suspects septic arthritis and is planning urgent surgical lavage, samples can be taken at the time of surgery and pre-operative aspiration may not be indicated. If the case is equivocal, a positive aspiration result may be helpful. Clinicians should not to be falsely reassured by a negative result. An aspirate containing crystals can help identify a crystal arthropathy but this does not exclude concurrent infection.^15,16^

If revision of a total joint is planned, isolation of an organism and obtaining sensitivities is desirable before committing to a single-stage revision as this can predict the potential success of the surgery.^12^ If a patient has received antibiotics he or she is less likely to have the responsible organism(s) isolated and may therefore be more likely to undergo a two-stage revision. Two-stage revision following unsuccessful irrigation and debridement has a higher failure rate and significant morbidity for the patient.^11^

Analysis of the causative organisms in this study confirmed the findings of previous studies^17^ with infections most commonly caused by either staphylococci or streptococci.^2,3,5,9,13,15^ In both the native and prosthetic knee there will be a number of cases caused by atypical pathogens where the standard antibiotics will be ineffectual. It may be that in these cases increasing the chance of isolating an organism is the most critical aspect of their management. It has been shown that failure of first line antibiotic regime is associated with a worse prognosis.^18^ Previous studies have shown that a delay in diagnosis and treatment leads to increased destruction of articular cartilage,^19^ and a worse prognosis in both the native^20^ and prosthetic knee.^14^ If clinicians are falsely reassured by false negative results, it may be that patients will have a delay before receiving appropriate treatment.

There are a number of limitations to the study. The first is that it is retrospective and so we do not have a comprehensive database of all aspirates in suspected cases that would have allowed us to calculate the specificity of the aspirate. It is not possible to evaluate the number of patients in the community treated successfully for superficial infections with antibiotics.

## Conclusions

Antibiotic administration should be avoided prior to joint aspiration in patients with a suspected diagnosis of septic arthritis of the knee joint. Clinicians should take an accurate history of the administration of antibiotics prior to presentation. This needs to be taken into account when interpreting the results of joint aspiration so the clinician is not falsely reassured by negative microscopy and Gram staining.
